# Modulatory influence of *Acacia hydaspica* R. Parker ethyl acetate extract against cisplatin inveigled hepatic injury and dyslipidemia in rats

**DOI:** 10.1186/s12906-017-1824-y

**Published:** 2017-06-12

**Authors:** Tayyaba Afsar, Suhail Razak, Ali almajwal, Muhammad Rashid khan

**Affiliations:** 1 Department of Biochemistry, Faculty of Biological Sciences, Quaid-i-Azam University, Islamabad, Pakistan; 2 Department of Animal Sciences, Faculty of Biological Sciences, Quaid-i-Azam University, Islamabad, Pakistan; 3 Department of Community Health Sciences, College of Applied Medical Sciences, King Saud University, Riyadh, Kingdom of Saudi Arabia

**Keywords:** Cisplatin, Lipid profile, Antioxidant, Oxidative stress, Liver fibrosis, Hepatotoxicity

## Abstract

**Background:**

Cisplatin (CP) is recommended as a first-line chemotherapeutic agent for solid tumors, however its usage outcomes in severe adverse effects. *Acacia hydaspica* possesses various phytochemicals and pharmacological activities. The current study aimed to investigate the protective effect of *A. hydaspica* ethyl acetate extract (AHE) against CP induced aberrations in lipid profile and hepatotoxicity.

**Methods:**

Rats were randomly separated into six groups (*n* = 6). Group 1 (control) received distilled water orally for 21 days. Groups 2 (CP control) received a single dose of CP (7.5 mg/kg bw, i.p) on day 16, group 3 (Plant control) received AHE (400 mg/kg b.w, oral) for 21 days, group 4 (post treated group); CP received on day 16 and AHE (400 mg/kg b.w/day, p.o.) was administered after CP till day 21, Group 5 (pretreated group) received AHE (400 mg/kg b.w/day, p.o.) for 21 days and CP (7.5 mg/kg b.w., i.p.) on day 16, group 6 (Silymarin + CP) received 100 mg/kg b.w., p.o. (11 doses/21 days) and CP (7.5 mg/kg b.w., i.p.) on day 16. Lipid profile, liver functional tests, oxidative stress markers, antioxidant enzymes status and histopathological changes were examined.

**Results:**

The present study revealed that CP caused body weights loss and increase liver index. CP significantly increased serum total lipid, triglycerides and LDL-cholesterol levels. Conversely, it significantly decreased serum HDL-cholesterol level. CP induced marked deteriorations in serum liver function biomarkers, reduced antioxidant enzymes in tissue, while elevated tissue oxidative stress markers along with morphological injuries compared to control rats. Treatment with AHE ameliorated CP induced alterations in lipid profile, serum ALT, AST, ALP and total bilirubin levels and liver weight. Furthermore AHE treatment improved the total protein and antioxidant enzymes levels while decreased the level of MDA, H_2_O_2_, and NO. The altered parameters were returned to the control level with AHE pretreatment. Histopathological analysis also supported the biochemical findings. Pretreatment seems to be more effective compared to post treatment indicating protective effect.

**Conclusion:**

These results reveal that treatment of AHE may be useful in the prevention of CP induced hepatotoxicity due to its antioxidant potential and polyphenolic constituents.

## Background

Several drugs of the modern pharmacopoeia induce liver injury with different clinical expositions. Liver impairment due to chemotherapy ensues often in an unpredictable or idiosyncratic manner. In the most severe cases, drug-persuaded liver injury usually require liver transplantation or lead to demise of the patient [1]. Even though remarkable advances occur in understanding the mechanisms of action and interconnection amongst the liver and chemotherapy; the underlying etiology of hepatic toxicity remains obscure [2].

Cisplatin (cis diamminedichloroplatinum-II, CP) is a widely recommended drug as a front line adjuvant therapy in “battle against cancer” such as testicular, gut, stomach, head and neck, ovarian, cervical, germ cell tumors, non-small cell lung carcinoma etc. The cytotoxic and apoptotic effects of CP are not exclusively impelled in cancer cells but destroy normal dividing cells as well, thus CP treatment is overwhelmed by various problems including drug resistance and detrimental side effects such as; nephrotoxicity, bone marrow suppression, gastrointestinal toxicity, neurotoxicity, ototoxicity and hepatotoxicity [3, 4].

Hepatotoxicity is still less considered perspective in the course of CP chemotherapy and CP has been rarely inspected as hepatic-toxicant. However, the hepatic drug metabolism is severely abnormal in investigational animals and humans with CP induced renal impairments. As CP treatment outcomes in acute renal failure (ARF) which is associated with an isozyme selective abnormal regulation of hepatic cytochrome P450 enzyme together with down-regulation of specific hepatic microsomal and male specific P450 isozymes in rats. Hence the down regulation of hepatic P450 enzymes in male rats after CP treatment is specifically accompanying an impairment of hepatic and testicular functions [5, 6].

Liver play a vital role in lipid metabolism and transport. Dyslipidemia and hepatic steatosis are considered as adverse effects resulted from CP chemotherapy. If the chemotherapeutic treatment is not discontinued, steatosis can advance towards steatohepatitis, which is epitomized not only by lipid build-up but also by necro-inflammation and fibrosis. Oxidative stress induced lipid peroxidation, alter tissue thiol status, protein denaturation, DNA damage, inflammation and apoptosis of normal cells with concomitant decrease in enzymatic and non-enzymatic antioxidant systems are considered the major deteriorations in CP induced toxicity [7, 8]. Moreover, functional and structural mitochondrial injury, apoptosis, trepidation in Ca2+ homeostasis [9] and inducible nitric oxide synthase (iNOS) may play significant role in CP induced liver toxicity [10]. Since CP remains one of the most efficacious antineoplastic drugs employed in chemotherapy, approaches to secure tissues from CP detrimental effects are of clinical interest [11].

Medicinal plants have been exploited since eras for maintaining health and treating diseases prior to the advent of modern medicines [12]. Natural or dietary compounds retain little or no toxicity could be used solely or in combination with conventional chemotherapeutic agents to treat various malignancies [13]. Various medicinal plants were investigated for their protective abilities against cisplatin-induced hepatotoxicity in animal models [14, 15]. Species from genus *Acacia* are one of the richest source of bioactive flavonoids, alkaloids, phenolics, saponins, polysaccharides, tannins, and terpenoids [16]. *Acacia hydaspica* R. Parker family Leguminosae is medicinally important plant. The bark and seeds are rich in tannins. Plant is commonly used as fodder [17, 18]. *A. hydaspica* possess antioxidant, anti-inflammatory, antidepressant, anticancer activity [19–21]. *A. hydaspica* possess various bioactive metabolites i.e. 1,2-Benzenedicarboxylic acid mono (2-ethylhexyl) ester, α-Amyrin, Vitamin E, 2,6-dimethyl-N-(2-methyl-à-phenylbenzyl) aniline and Squalene were detected by GCMS analysis of methanol extract [22], while gallic acid catechin, rutin, caffeic acid were identified by HPLC analysis of methanol extract. Ethyl acetate fraction of *A. hydaspica* showed excellent anti-inflammatory potential in rats and antioxidant activity in vitro. HPLC analysis indicated the occurrence of gallic acid, catechin and myricitin in AHE, while bioactivity guided phytochemical investigation led to the isolation of some polyphenolic compounds i.e., 7-*O*-galloyl catechin, +catechin and methyl gallate as anticancer compounds from ethyl acetate fraction of *A. hydaspica;* as major anticancer compounds against breast and prostate cancer cell lines [20, 21].Various species of genus *Acacia* were investigated for their antioxidant and hepatoprotective potentials in animal models [23]. Polyphenol rich fraction of *A. arabica* is a potent free radical scavenger and hepatoprotective against CCl _4_-induced hepatic damage. These activities were ascertained due to the presence of (+) catechin, (−) epicatechin, (+) dicatechin, quercetin, gallic acid, (+) leucocyanidin gallate, sucrose and (+) catechin-*5*-gallate in the bark extract [24]. *A. nilotica* bark extract prevents hepatic malondialdehyde induction and reduces liver injury [25]. *A. nilotica* bark extract enhance the antioxidant enzymes functioning and deteriorate oxidative stress in the hepatic tissue of N-nitrosodiethylamine treated rats [16]. Kannan and colleagues described the hepatoprotective effect of *A. nilotica* against acetaminophen-induced hepatic injury in wistar rats [26]. Another study reported the hepatoprotective potential of *A. senegal* pods extract against CCl_4_ induced liver injury in rats [27]. The ethanol and aqueous extracts of *A. ferruginea* leaves showed their efficacy against CCl_4_ induced hepatotoxicity in Wistar albino rats [28]. The occurrence of bioactive constituents i.e. flavonoids and tannins, may offer hepatoprotection.

Based on hepatoprotective potential of related species in animal models and varied in vitro pharmacological properties of *A. hydaspica*, in this study an attempt was made to study its hepatoprotective activity against the cisplatin-induced liver injury. Various biochemical parameters and histological examination was performed to apprehend the protective potential of plant extract against CP induced liver injury.

## Methods

### Plant collection

The aerial parts (bark, twigs, and leaves) of *A. hydaspica* were collected from Kirpa charah area Islamabad, Pakistan. Plant specimen was identified by Dr. Sumaira Sahreen (Curator at Herbarium of Pakistan, Museum of Natural History, Islamabad). A voucher specimen with Accession No. 0642531 was deposited at the Herbarium of Pakistan, Museum of Natural History, Islamabad for future reference.

### Drug and plant dose preparation

Cisplatin (CP) injection (Sigma-Aldrich, St. Louis, MO, U.S.A.) was dissolved in saline and 7.5 mg/kg body weight dose of CP was selected on the basis of previous literature to induce testicular toxicity [29]. *A. hydaspica* methanol extract was fractionated as previously described [30], and its ethyl acetate extract (AHE) (the most bioactive extract under in vitro examinations and containing bioactive polyphenols [31] was selected for further in vivo investigation. Silymarin (100 mg/kg b.w) and AHE (400 mg/kg b.w) were freshly prepared in distilled water before dosing. The dose of extract was selected based on our pilot experiment.

### Acute toxicity evaluation

The acute toxicity study was conducted as per the guidelines 425 of the Organization for Economic Cooperation and Development (OECD) for testing of chemicals for acute oral toxicity [32]. Male rats (*n* = 6) were treated with different doses (50, 250, 500, 1000, 2000, 3000 and 4000 mg/kg, p.o.) of AHE, while the control group received saline (10 ml/kg). Animals were observed continuously for 2 h for behavioral, neurological and autonomic profiles and after a period of 24 and 72 h for any lethality or death [31]. One forth (400 mg/kg b.w.) of maximum tested dose was selected on the basis of our pilot study for further experiments.

### Experimental design

Forty (40) male Sprague Dawley rats (200–225 g) from Primate Facility at Quaid-i-Azam University, Islamabad were kept in ordinary cages at room temperature of 25 ± 3 °C with a 12 h dark/light cycle in pathogen free environment. They were allowed to standard laboratory feed and water. Guidelines of national institute of animal health (NIH guidelines) were strictly adapted for experimentations. The ethical board of Quaid-i-Azam University, Islamabad permitted the experimental protocol (Bch#264). The experimental plan was designed according to previous studies [33] with minor modifications. Animals were distributed into six groups (*n* = 6). The following treatment procedure was adopted for the study.

Group I: Control received water for 21 days, and saline injection (2 ml/kg, i.p) on day 16.

Group II: CP treated; received one dose of CP (7.5 mg/kg b.w., i.p.) on day 16th of experiment for inducing organ toxicity, and distilled water for 21 days (oral).

Group III: AHE treated; 400 mg/kg body weight/day oral dose for 21 days

Group IV: CP + AHE (post treated group); CP on day 16 and AHE (400 mg/kg b.w/day, p.o.) was administered from day 16 to 21.

Group V: AHE + CP (pretreated group); received 400 mg/kg body weight/day, p.o. for 21 days and CP (7.5 mg/kg b.w., i.p.) on day 16.

Group VI: Silymarin + CP; received 100 mg/kg b.w., p.o. dose every other day (11 doses/21 days) and CP (7.5 mg/kg b.w., i.p.) on day 16.

At the end of the experiment, animals were weighed and sacrificed. Trunk blood was taken with 23 G1 syringes and collected in sterile falcon tubes. Blood was centrifuged at 500×g for 15 min at 4 °C to obtained serum and kept at −80 °C until biochemical analysis**.** Liver tissues were dissected out, wash with saline and weigh immediately. The liver index was calculated according to the formula;

$$ \left[\frac{liver\  weight}{rat\  weight}\right]\times 100 $$

Subsequently, half of the organ was treated with liquid nitrogen and stored at -80 °C for further biochemical analysis while the other portion was processed for histology [26].

### Estimation of serum markers of liver function and dyslipidemia

Serum analysis of various liver marker enzymes such as alanine aminotransferase (ALT), aspartate aminotransferase (AST), alkaline phosphatase (ALP), lactate dehydrogenase (LDH) and lipid profile; level of total cholesterol (TC), high-density lipoproteins (HDL), low-density lipoproteins (LDL) and triglycerides (TG) were estimated by using standard AMP diagnostic kits (Stattogger Strasse 31b 8045 Graz, Austria).

#### Biochemical analysis

##### Homogenate preparation

100 mg of each liver tissues sample was homogenized in 10 volume of 100 mM KH_2_PO_4_ buffer containing 1 mM EDTA, pH 7.4. The homogenate was centrifuged at 12000×g for 30 min at 4 °C to remove cell debris and the supernatant was saved in aliquots and stored at −20 °C for assaying antioxidant enzymes, lipid peroxidation products, H_2_O_2_ and nitrite content.

##### Estimation of tissue protein content

Lowry et al. procedure was followed to estimate the total soluble proteins within the tissue samples [34]. Tissue sample (100 mg) was homogenized in potassium phosphate buffer. Homogenized mixture was centrifuged at 4 °C at 10000×g for 15–20 min to obtain the supernatant. Alkaline solution 1 ml was added in 0.1 ml of supernatant and vortexed. Then the incubation was done for 30 min. Afterwards the change in absorbance was calculated at 595 nm. Bovine serum albumin (BSA) standard calibration curve was used to find out the concentration of serum proteins in the sample.

#### Measurement of tissue antioxidant status

##### Catalase (CAT) activity

CAT activity was determined by the protocol of Khan et al. with slight modifications [35]. The CAT reaction solution consists of 625 μl of 50 mM of potassium phosphate buffer (pH 5), 100 μl of 5.9 mM H_2_O_2_ and 35 μl enzyme extract. Change in the absorbance of the reaction solution was noted after 1 min at 240 nm. An absorbance change of 0.01 as units/min denotes one unit of catalase activity.

##### Peroxidase (POD) activity

Activity of POD was assayed by previously described protocol with slight modifications [35]. POD reaction solution contains 40 mM hydrogen peroxide (75 μl), 20 mM guaiacol (25 μl) and 625 μl of 50 mM potassium phosphate buffer (pH 5.0) and 25 μl of tissue homogenate. After an interval of one minute, change in absorbance was determined at 470 nm. One unit POD activity is equivalent to change in absorbance of 0.01 as units/min.

##### Superoxide dismutase (SOD) activity

Kakkar et al. method was utilized for the assessment of SOD activity [35]. Phenazine methosulphate and sodium pyrophosphate buffers were exploited for the assessment of SOD activity. Centrifugation of tissue homogenate was done at 1500×g for 10 min and then at 10,000×g for 15 min. Supernatant was collected and 150 μl of it was added to the aliquot containing 600 μl of 0.052 mM sodium pyrophosphate buffer (pH 7.0) and 186 mM of phenazine methosulphate (50 μl). 100 μl of 780 μM NADH was added to initiate enzymatic reaction. After 1 min, glacial acetic acid (500 μl) was added to stop the reaction. At 560 nm optical density was determined to enumerate the color intensity. Results were evaluated in units/mg protein.

##### Quinone reductase assay (QR)

The Quinone reductase activity in tissues of different treatment groups was evaluated as described earlier [36]. Reaction mixture in a total volume of 3 ml comprised of 25 mM Tris-HCl buffer (2.13 ml; pH 7.4), 700 μl of BSA, 100 μl of FAD, 20 μl of 0.1 mM NADPH and 100 μl of tissue homogenate. Reduction of dichlorophenolindophenol (DCPIP) was noted at 600 nm. Enzyme potency was estimated as nM of DCPIP reduced/min/mg protein by employing molar extinction coefficient of 2.1 × 10^4^/M/cm.

##### Reduced glutathione (GSH) estimation test

Reduced glutathione activity was checked as described by Jollow [37]. Precipitation of tissue homogenate (500 μl) was carried out by the addition of (500 μl) 4% sulfosalicylic acid. 1 h of incubation at 4 °C was done then the samples were centrifuged for 20 min at 1200×g. An aliquot of 33 μl supernatant was collected and added to aliquots consisting of 900 μl of 0.1 M potassium phosphate buffer (pH 7.4) and 66 μl of 100 mM DTNB. Reaction of GSH with DTNB produced a yellow colored complex reduced glutathione. Through spectrophotometer absorption was observed at 412 nm. The GSH activity was measured by μM GSH/g tissue.

##### Glutathione-S-transferase (GST)

Scheme of Habig et al. [38] was followed for the estimation of GST potency. The assay principle relies on the formation of CDNB conjugate. 150 μl aliquot of tissue homogenate was added to 720 μl of sodium phosphate buffer together with 100 μl of reduced glutathione (1 mM) and 12.5 μl of CDNB (1 mM). By spectrophotometer, optical density was recorded at 340 nm. Through molar coefficient of 9.61 × 10^3^/M/cm GST activity was estimated as amount of CDNB conjugate formed per minute per mg protein.

##### Glutathione reductase assay (GR)

Glutathione reductase activity in tissue samples was analyzed as described by Carlberg and Mannervik [39]. The reaction reagent 2 ml was made of of 1.65 ml phosphate buffer: (0.1 M; pH 7.6), 100 μl EDTA (0.5 mM), 50 μl oxidized glutathione (1 mM), 100 μl NADPH (0.1 mM) and 100 μl of homogenate. Activity of enzyme was monitered by recording the absorbance of the vanishing of NADPH at 340 nm at 25 °C. Estimated of enzyme level was accomplished as nM NADPH oxidized/min/mg protein by employing molar extinction coefficient of 6.22 × 10^3^/M/cm.

##### Glutathione peroxidase assay (GPx)

Glutathione peroxidase activity was assessed as described elsewhere [40]. Entire volume of 2 ml reaction solution comprised of 1 mM EDTA (100 μl), 0.1 M phosphate buffer (1.49 ml; pH 7.4), 1 m M sodium azide (100 μl), 1 IU/ml glutathione reductase (50 μl), 1 mM GSH (50 μl), 0.2 mM NADPH (100 μl), 0.25 mM H_2_O_2_ (10 μl) and tissue homogenate (100 μl). The loss of NADPH was recorded at 340 nm at room temperature. Enzyme level was estimated as nM NADPH oxidized/min/mg protein employing 6.22 × 10^3^/M/cm molar extinction coefficient.

##### γ-Glutamyl transpeptidase (γ-GT)

The activity of γ-glutamyl transpeptidase was checked following Orlowski et al. scheme [41]. Glutamyl nitroanilide was used as substrate for verification of the activity of γ-GT. Reaction solution of γ-GT consist of an aliquot of 50 μl tissue homogenate, 250 μl of glutamyl nitroanilide (4 mM), 250 μl of glycyl glycine (40 mM) and 250 μl of MgCl2 (11 mM) which was prepared in 185 mM Tris HCl buffer at room temperature. After 10 min of incubation, the reaction was stopped with the addition of 250 μl 25% trichloro acetic acid. Then centrifugation was done at 2500×g for 10 min and the optical density of collected supernatant was determined at 405 nm. Activity of γ-GT was determined as nM nitroaniline formed per min per mg protein by the aid of molar extinction coefficient of 1.75 × 10^3^/M/cm.

#### Measurement of oxidative stress markers in liver

##### Lipid peroxidation assay (MDA content)

Protocol of Iqbal et al. [42] was adopted with slight modifications for the assessment of lipid peroxidation. The reaction mixture consists of 0.1 M phosphate buffer of 290 μl (pH 7.4), 100 mM ferric chloride (10 μl), 100 mM ascorbic acid (100 μl) and 100 μl of homogenized sample. For 1 h incubation of the solution was done in shaking water bath at 37 °C. 10% trichloroacetic acid (500 μl) was added to inhibit the reaction. At that point 0.67% thiobarbituric acid (500 μl) was poured and the reaction tubes were stayed for 20 min in the water bath. After that the tubes were placed in crushed ice bath for 5 min and centrifugation was done at 2500×g for 12–15 min. By spectrophotometer absorbance was calculated at 535 nm counter to a reagent blank. By exploiting the molar extinction coefficient of 1.56 × 10^5^/M/cm. Results were estimated as nM of TBARS generates per min per mg tissue at a temperature of 37 °C.

##### Hydrogen peroxide assay

Estimation of hydrogen peroxide activity in tissue samples was monitored by method described earlier [43]. In the reaction mixture, 500 μl of 0.05 M phosphate buffer (pH 7), 100 μl of homogenate was added along with 100 μl of 0.28 nM phenol red solution, 250 μl of 5.5 nM dextrose and horse radish peroxidase (8.5 units) was added. Incubation was done at room temperature for 60 min. 100 μl of NaOH (10 N) was added to stop the reaction. Then mixture tubes were centrifuged for 5–10 min at 800×g. The absorbance of the supernatant was calculated against reagent blank at 610 nm. Production of H_2_O_2_ was measured as nM H_2_O_2_/min/mg tissue by employing the standard curve of phenol red oxidized by H_2_O_2_.

##### Nitrite assay

For the execution of nitrite assay, Griess reagent was utilized [44]. Briefly, tissue samples (100 mg each) were de-proteinised in 100 μl solution comprising 5% ZnSO_4_ and 0.3 M NaOH. Samples were Centrifuge at 6400×g for 15–20 min. Remove supernatant and add 20 μl in a cuvette containing 1 ml of Griess reagent, change in color was determined at 540 nm. Griess reagent 1 ml was used as a blank in the spectrophotometer (Smart Spec TM Spectrophotometer). Standard curve of sodium nitrite was utilized for the quantification nitrite concentration in testicular tissues.

#### Histopathological examination by light microscopy

For histopathological examination, hepatic tissues from each group were fixed in a fixative containing absolute alcohol (85 ml), glacial acetic acid (5 ml) and 40% formaldehyde (10 ml). After dehydration tissue samples were fixed in parafin to prepare blocks for microtomy. Tissues were sectioned 4–5 μm with microtome and stained with Hemotoxilin-Eosin (H&E) and studied under a light microscope (DIALUX 20 EB) at 40X. Photographs were taken with same the zoom and the camera settings were used and histological parameters were analyzed.

### Statistical analysis

Data are expressed mean ± SEM (*n* = 6). One way analysis of variance (ANOVA) followed by Tukey’s test was used for analyzing the statistical differences between different treatment groups using Graph pad prism 5 software. Level of significance was set at *p* < 0.05

## Results

### Acute toxicity evaluation

Since toxicity of the test sample was a major concern, hence acute toxicity evaluation was done before proceeding to in vivo experiment. AHE was found to be safe at all tested doses (up to 4000 mg/kg b.w) and it did not induce any detrimental indications in rats like sedation, convulsions, diarrhea and irritation. During the 3 days of the assessment, no mortality was observed.

### AHE treatment is not associated with toxicity

**Fig. 1:**
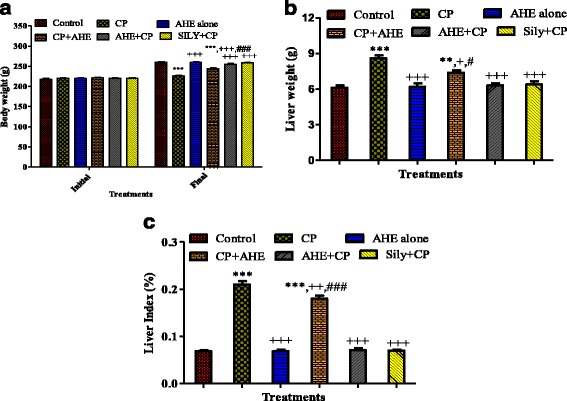
Effect of the ethyl acetate extract of *A. hydaspica (AHE)* (400 mg/kg·bw) on **a** body weight, **b** liver weight and **c** liver index (%) in various treatment groups. Mean ± SE (*n* = 6); statistical symbols in 1 a indicated significant difference in final vs initial body weight; in 1b and 1c ^*, **, ***^ indicated significance from the control group at *p* < 0.05, *p* < 0.01 and *p* < 0.0001 probability level, ^+, ++, +++^ indicate significance from the CP group at *p* < 0.05, *p* < 0.01 and *p <* 0.0001 while ^##^ indicate significance of AHE pre-treatment vs post treatment group at *p* < 0.01 probability level **(**One way ANOVA followed by Tukey’s multiple comparison tests)

In all treatment groups no clinical signs of toxicity (such as, unusual salivation, flick-king movements, shiver, head and forelimb clonuses, spasms, incoordination, diarrhea and increased diuresis) were observed. No death was witnessed in any treatment group during the experimental period. The animals treated with AHE showed similar body weight gain compared to the control group (Fig. 1a). Similarly, no significant change in liver weight was recorded in AHE and control groups (Fig. 1b), suggesting that there was no toxicity associated with AHE dosage. The hepatic tissues from either control or AHE treated rats revealed no obvious variations in histoarchitecture (Fig. 3; group 1 and 3).

### Effect of AHE on body weight and liver index

CP treatment caused significant reduction (*p* < 0.0001) in body weight while increased the absolute and relative liver weight (liver index) in comparison to control group. These alterations were significantly (*p* < 0.05) and (*p* < 0.0001) restored with post and pretreatment of AHE (400 mg/kg, b.w) respectively (Fig. 1a, b & c). AHE pretreatment showed similar results as silymarin treated animals.

### Protective effect of AHE on lipid profile

The protective effect of AHE on lipid profile such as total cholesterol, HDL, LDL and triglycerides is summarized in Table 1. CP markedly (*p* < 0.001) increased the levels of total cholesterol, LDL and triglycerides, whereas the HDL levels were significantly (*p* < 0.001) decreased in CP group. Administration of AHE with CP markedly (*p* < 0.001) corrected the serum markers of lipid profile, with most significant (*p* < 0.001) protection observed in AHE pre-treatment group in comparison to post-treatment group. AHE pre-treatment results in an influence equal to silymarin treated group.

**Table 1:** Effect of cisplatin (CP) and different treatments of AHE on lipid profile

Group	Total cholesterol (mg/dl)	HDL (mg/dl)	LDL (mg/dl)	Triglycerides (mg/dl)
Control	76.50 ± 0.29 ^b^	25.17 ± 0.44^b^	48.62 ± 0.23^b^	70.50 ± 1.04^b^
CP	95.63 ± 0.32 ^a^	20.30 ± 0.36^a^	165.4 ± 0.32^a^	92.83 ± 0.60^a^
AHE alone	76.13 ± 0.19 ^b^	25.33 ± 0.33^b^	48.57 ± 0.20^b^	70.33 ± 0.67^b^
CP + AHE	90.33 ± 0.33 ^a, b, d^	20.83 ± 0.44^a, d**^	144.0 ± 0.58^a, b^	84.67 ± 0.67^a, b, d^
AHE + CP	79.83 ± 0.44 ^a, b, c^	24.03 ± 0.15^b, c**^	59.67 ± 0.33^a, b^	76.30 ± 0.35^a, b, c^
CP + Sily	78.67 ± 0.33 ^a**, b^	24.17 ± 0.61^b^	58.43 ± 0.22^a, b^	73.50 ± 0.50^b^

Values expressed as mean ± SEM

^a^ Significance at *p* < 0.0001 Vs. control group

^b^ Significance at *p* < 0.0001 Vs. Cisplatin (CP) group

^c^ Significance at *p* < 0.0001 of AHE + CP pre-treated group Vs. CP + AHE post-treated group

^d^ Significance at *p* < 0.0001 of CP + AHE treatment groups Vs CP + Sily group

^**^ Significant difference *p* < 0.001. Non-significant difference (*p* > 0.05) was recorded between control and AHE alone treated group in all parameters (One way ANOVA followed by Tukey’s multiple comparison tests). Sily; Silymarin

### Protective effect of AHE against cisplatin induced hepatotoxicity

#### Effect of AHE on serum biomarkers of liver function

**Fig. 2:**
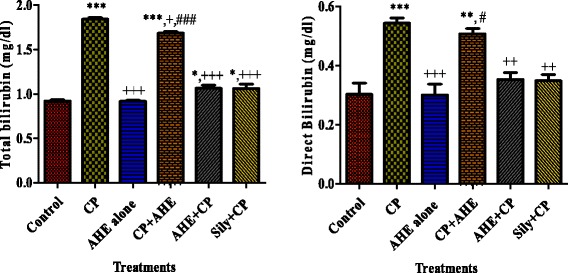
Effect of cisplatin (CP) and different treatments of AHE on serum total bilirubin and direct bilirubin profile, Values expressed as mean ± SEM. ^*, **, ***^ shows significance from control group at *p* < 0.05, *p* < 0.001, *p* < 0.0001, ^++, +++^ shows significance from Cisplatin (CP) group at *p* < 0.001, *p* < 0.0001. #, ### shows significance from AHE + CP pre-treated group vs. CP + AHE post-treated group respectively. Non-significant difference (*p* > 0.05) was recorded between control and AHE alone treated group in all parameters (One way ANOVA followed by Tukey’s multiple comparison tests)

The estimation of serum biomarker enzymes is a supportive index to analyze the hepatic functionality and all types of hepatocellular lesions. CP inoculation induced liver damage as shown by significantly (*p* < 0.001) elevated levels of hepatic biomarkers in serum viz., aminotransferase enzymes (ALT and AST), especially ALT is a specific and key indicator of liver harm, alkaline phosphatase (ALP), lactate dehydrogenase (LDH) (Table 2), total serum bilirubin (TSB) and direct bilirubin (DB) (Fig. 2) levels were also augmented in contrast to those of control rats. Table 2 and Fig. 2 exhibited the recovery pattern of AHE pre- and post-treatment groups on hepatic function biomarkers of serum in CP induced hepatocellular injury. AHE + CP treatment significantly decreased the elevated levels of ALT, AST, ALP, LDH, TSB and DB as compared to only CP treated rats and CP + AHE group. Pre-treatment of rats with AHE prevent CP deteriorations and maintained the hepatic serum biomarker near control group and their values were non-significantly different from reference drug silymarin.

**Table 2:** Effect of cisplatin (CP) and different treatments of AHE on liver biomarkers in serum

Group	ALT (U/l)	AST (U/l)	ALP (U/l)	LDH (U/l)
Control	44.63 ± 0.49 ^b^	72.83 ± 0.12^b^	120.7 ± 0.33 ^b^	70.50 ± 1.04^b^
CP	138.3 ± 0.29^a^	192.4 ± 0.35^a^	350.0 ± 0.58 ^a^	92.83 ± 0.60^a^
AHE alone	44.57 ± 0.23^b^	72.63 ± 0.19 ^b^	120.9 ± 0.21^b^	70.33 ± 0.67^b^
CP + AHE	101.8 ± 0.28^a, b, d^	140.4 ± 0.30 ^a, b, d^	299.2 ± 0.42^a, b, d^	84.67 ± 0.67^a, b, d^
AHE + CP	46.60 ± 0.31^a, b, c^	75.97 ± 0.55^a, b, c^	135.8 ± 0.28^a, b, c^	76.30 ± 0.35^a, b, c^
CP + Sily	47.17 ± 0.17^a**, b^	75.53 ± 0.53 ^a, b^	134.0 ± 0.55^a, b^	73.50 ± 0.50^b^

Values expressed as mean ± SEM

^a^ Significance at *p* < 0.0001 Vs. control group

^b^ Significance at *p* < 0.0001 Vs. Cisplatin (CP) group

^c^ Significance at *p* < 0.0001 of AHE + CP pre-treated group Vs. CP + AHE post-treated group

^d^ Significance at *p* < 0.0001 of CP + AHE treatment groups Vs CP + Sily group

^**^ Significant difference at *p* < 0.001. Non-significant difference (*p* > 0.05) was recorded between control and AHE alone treated group in all parameters (One way ANOVA followed by Tukey’s multiple comparison tests)

#### Effect of AHE on hepatic antioxidant enzymatic status and GSH profile

CP therapy leads to generation of reactive oxygen metabolites and inhibited the action of antioxidant enzymes and lipid peroxidation. Mechanism of CP induced hepatic toxicity is possibly the depletion of enzymatic antioxidants especially reduced glutathione (GSH), significant elevation in hepatic MDA levels and increased oxidative stress. Antioxidants are very crucial for the detoxification of reactive metabolites and maintaining cellular balance. Table 3 indicates significant decrease in phase I antioxidant enzymes of liver i.e., POD, SOD, CAT and QR levels following single dose of CP as compared to untreated control and AHE alone treated groups. Prophylactic treatment with AHE before and after CP administration significantly restored the activity of these enzymes while in AHE pre-treatment group the levels of enzymes were non-significantly different from standard drug (silymarin) group. Oral administration of AHE alone (at 400 mg/kg) exhibited non-significant alteration in the efficacy level of these antioxidant enzymes in comparison to control values.

**Table 3:** Effect of cisplatin (CP) and different treatments of AHE on Phase I antioxidant enzymes

Group	POD (U/min)	SOD (U/mg protein)	CAT (U/min)	QR (nM/min/mg protein)
Control	13.40 ± 0.23^b^	1.55 ± 0.06^b^	24.02 ± 0.08 ^b^	104.8 ± 1.01^b^
CP	7.23 ± 0.39^a^	0.39 ± 0.03^a^	12.80 ± 0.05 ^a^	67.8 ± 0.88 ^a^
AHE alone	13.09 ± 0.27^b^	1.55 ± 0.08^b^	24.03 ± 0.03 ^b^	104.7 ± 1.07 ^b^
CP + AHE	9.21 ± 0.29^ab*,d^	1.08 ± 0.05^a, b^	14.70 ± 0.06 ^a, b, d^	82.2 ± 0.89 ^a, b,d^
AHE + CP	12.50 ± 0.66^b, c**^	1.30 ± 0.03^a*, b^	20.37 ± 0.08 ^a, b, c^	95.3 ± 0.65 ^a, b, c^
CP + Sily	12.57 ± 0.44^b^	1.31 ± 0.04^a*, b^	20.66 ± 0.04 ^ab^	95.9 ± 1.26 ^a,b^

Values expressed as mean ± SEM

^a^ Significance at *p* < 0.0001 Vs. control group

^b^ Significance at *p* < 0.0001 Vs. Cisplatin (CP) group

^c^ Significance at *p* < 0.0001 of AHE + CP pre-treated group Vs. CP + AHE post-treated group

^d^ Significance at *p* < 0.0001 of CP + AHE treatment groups Vs CP + Sily group

^*,**^ Significant difference at *p* < 0.001. Non-significant difference (*p* > 0.05) was recorded between control and AHE alone treated group in all parameters (One way ANOVA followed by Tukey’s multiple comparison tests)

Table 4 shows the protective effect of AHE on phase II antioxidant enzymes including QR, GSH, GR, GST, γ-GT and GPx in the liver tissue of rat. CP single dose leads to marked (*p* < 0.001) deterioration in the activity of hepatic phase II antioxidant enzymes. The presence of AHE prior to or after CP injection attenuated the CP intoxication and significantly (*p* < 0.001) restored the enzyme activity, however the treatment seems more effective (*p* < 0.0001) when AHE was administered prior to CP inoculation. AHE alone treated group showed non-significant change in phase II antioxidant enzymes activity in comparison to that of control.

**Table 4:** Effect of cisplatin (CP) and different treatments of AHE on phase II antioxidant enzymes

Group	GSH (μM/g tissue)	GR (nM/min/mg protein)	GST (nM/min/mg protein)	γ-GT (nM/min/mg Protein)	GPx (nM/min/mg Protein)
Control	19.61 ± 0.28 ^b^	163.6 ± 0.65 ^b^	142.6 ± 0.57 ^b^	336.2 ± 0.56 ^b^	139.5 ± 1.16 ^b^
CP	10.08 ± 0.28 ^a^	103.9 ± 0.65 ^a^	101.4 ± 0.84 ^a^	143.6 ± 1.44 ^a^	90.53 ± 1.71 ^a^
AHE alone	20.11 ± 0.16 ^b^	164.2 ± 0.68 ^b^	142.7 ± 0.89 ^b^	336.3 ± 1.0 ^b^	139.8 ± 0.36 ^b^
CP + AHE	12.83 ± 0.21^a,b d^	122.8 ± 0.65^a,b,d^	122.1 ± 0.87^a,b,d^	215.3 ± 1.85^a,b,d^	108.6 ± 1.25 ^a,b^
AHE + CP	18.38 ± 0.29^a*,b, c^	145.5 ± 0.58^a,b,c^	135.3 ± 0.77^a,b, c^	310.4 ± 0.6 ^a,b,c^	128.6 ± 1.74 ^a,b^
CP + Sily	18.34 ± 0.11^a*,b^	148.7 ± 1.09 ^a, b^	130.0 ± 0.92 ^a, b^	309.5 ± 0.69 ^a, b^	131.8 ± 1.28 ^a,b^

Values expressed as mean ± SEM

^a^ Significance at *p* < 0.0001 Vs. control group

^b^ Significance at *p* < 0.0001 Vs. Cisplatin (CP) group

^c^ Significance at *p* < 0.0001 of AHE + CP pre-treated group Vs. CP + AHE post-treated group

^d^ Significance at *p* < 0.0001 of CP + AHE treatment groups Vs CP + Sily group

^*^ Significant difference at *p* < 0.001. Non-significant difference (*p* > 0.05) was recorded between control and AHE alone treated group in all parameters (One way ANOVA followed by Tukey’s multiple comparison tests)

#### Effect of AHE on liver protein and oxidative stress markers

CP treatment resulted in substantial (*p* < 0.0001) decrease in liver tissue soluble protein as compared to control and AHE alone treated groups (Table 5). AHE significantly (*p* < 0.0001) ameliorated the toxicity and restored tissue protein content in both pre-treatment and post-treatment regimes in comparison to CP alone treated group, and maximum (*p* < 0.001) restoration was observed when AHE was presented before CP injection. The protein content recorded in case of AHE pre-treatment was non-significantly different to that of silymarin treated group.

Oxidative stress has been reflected as one of the underlying mechanism of CP-persuaded acute organ injury. Effect of pre and post-treatment of AHE on CP induced oxidative stress was evaluated by measuring the levels of H_2_O_2_, nitrite (NO) and lipid peroxidation products (MDA) (Table 5). CP-induced hepatic oxidative stress was noticeable by significant (*p* < 0.0001) intensification in H_2_O_2_, NO and MDA levels as compared to control and AHE alone treated groups. AHE pre-treatment bring about comprehensive diminution in the level of H_2_O_2_ and restored the normal values similar to that of control and silymarin treated group. The changes in nitrite content and MDA levels were significantly attenuated by AHE pre and post-treatments. The level of nitrite content and MDA formed in pre- and post-treatment groups were significantly different with *p* < 0.05 and *p* < 0.0001 respectively from control group, however pre-treatment with AHE produce equivalent effects to that of silymarin. AHE administration seems to be more effective in reducing the CP-induced oxidative stress by significantly (*p* < 0.0001) decreasing the level of H_2_O_2_, nitrite content and MDA formation (*p* < 0.05) in comparison to its administration after CP intoxication.

**Table 5:** Effect of cisplatin (CP) and different treatments of AHE on liver tissue protein, H_2_O_2_, nitrite content and lipid peroxidation

Group	Protein (μg/mg Tissue)	H_2_O_2_ (nM/min/mg Tissue)	Nitrite (NO) (content μM/ml)	MDA (nM/min/mg protein)
Control	4.66 ± 0.05^b^	1.97 ± 0.06^b^	34.77 ± 1.32^b^	3.5 ± 0.156^b^
CP	1.19 ± 0.05^a^	5.39 ± 0.256^a^	90.41 ± 1.28^a^	8.97 ± 0.337^a^
AHE alone	4.67 ± 0.15^b^	1.32 ± 0.06^b^	36.24 ± 0.96^b^	3.153 ± 0.087 ^b^
CP + AHE	3.25 ± 0.13^a,b,d**^	4.325 ± 0.198^a,b**,d^	71.48 ± 1.86^a,b,d^	6.11 ± 0.504^a,b, d*^
AHE + CP	3.92 ± 0.04^a**,b, c**^	2.38 ± 0.189^b,c^	41.09 ± 0.69^a*,b,c^	4.84 ± 0.06^a*,b,c*^
CP + Sily	3.93 ± 0.11^a**,b^	2.39 ± 0.037^b^	40.98 ± 1.38^a*,b^	4.90 ± 0.107^a*,b^

Values expressed as mean ± SEM

^a^ Significance at *p* < 0.0001 Vs. control group

^b^ Significance at *p* < 0.0001 Vs. Cisplatin (CP) group

^c^ Significance at *p* < 0.0001 of AHE + CP pre-treated group Vs. CP + AHE post-treated group

^d^ Significance at *p* < 0.0001 of CP + AHE treatment groups Vs CP + Sily group

^*,**^ Significant difference at *p* < 0.001. Non-significant difference (*p* > 0.05) was recorded between control and AHE alone treated group in all parameters (One way ANOVA followed by Tukey’s multiple comparison tests)

#### Histopathology assessment of liver

**Fig. 3:**
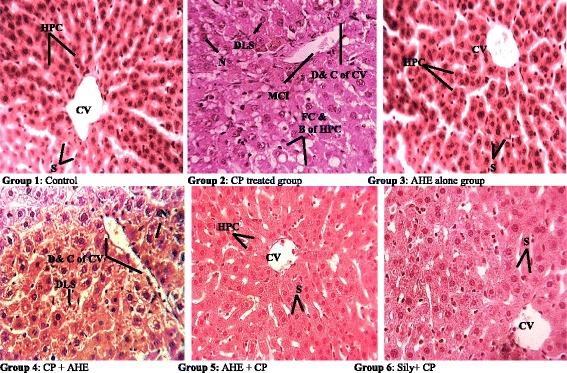
Histopathological effect of Cisplatin and protective effect of AHE in rat liver. (H&E staining, magnification 40X). Group 1: Liver section from control rats showing normal liver morphology. Group 2: CP-treated rat liver showing enhanced cellular lesions, loss of hepatic tissue structure arrangement and collection of inflammatory cells. Group 3: Represents liver section from AHE alone treated rat liver. Group 4: AHE post-treatment showed low density of cellular lesions. Group 5: AHE Pre-treatment results in significant protection against CP induced hepatic injury. Group 6: Showed protective effect of Silymarin treatment. AHE, *A. hydaspica* ethyl acetate fraction, CP-Cisplatin, HPC-Hepatocytes, CV-Central venule, MCI-Monocytes nuclear cells infiltrations, D&C-Dilation and congestion, FC & B- Fatty changes and ballooning, DLS- Degeneration of lobular shape, S-Sinosides, N-necrosis

Histopathological examination provides a significant support for the biochemical analysis. The microscopic inspection of liver tissue sections of control and AHE treated groups showed normal morphology (Fig. 3). The typical microscopic structural design of the liver was composed of hexagonal lobules and acini. Hexagonal lobules were centered on the central vein (CV) and have a portal triad comprising branches of the portal vein (PV), hepatic artery (HA) and bile duct (BD). The appearance of hepatocyte cord, hepatocytes, central vein and portal areas appeared normal in AHE alone and control groups. Hepatic structure of CP treated group displayed significant morphological alterations in hepatocytes lobular structure. CP single dose lead to macro-vesicular steatosis (fatty change), which occurs due to disrupted lipoprotein transport or accumulation of fatty acids; depicting CP might interferes with mitochondrial and microsomal function in hepatocytes leading to accumulation of lipids. Moreover CP administration induced austere cellular hypertrophy, necrosis, severe lobular degenerations or no discernable normal lobular architecture, interlobular passage became dilated, inflammatory cell infiltrations, wide vacuolar hepatocytes with ballooning degenerations, necrotic hepatocytes, dilation and congestion of central vein, pyknotic, excessive collagen deposition with disturbed epithelium.

Pre-treatment with AHE ameliorated CP induced hepatic injury; inhibit steatosis, necro-inflammatory alterations, fibrosis, lobular dilations, collagen deposition and vacuolization. While, retain the normal lobular architecture of hepatocytes and appearance of central vein. AHE post-treatment group showed less protection as compared to pre-treatment group in maintaining the normal hepatic morphology; steatosis and inflammatory cell infiltrations were obvious, however the severity of hepatic injury was markedly low in comparison to CP alone treated group. AHE pre-treatment provided similar level of protection as reference drug silymarin against CP deterioration. The histological observations supported the serological as well as biochemical findings.

## Discussion

Extensive number of investigations described the preventing potential of herbal products against the chemotherapy induced side effects [45]. We have hypothesized that AHE might be useful in attenuating CP-induced oxidative injury to hepatocytes due to its antioxidant activity and bioactive components. In both pretreatment and post treatment modules AHE was administered for 6 days after CP injection. In addition, to assess the effect of extract administration prior to CP injection, we have given rats AHE extract for 15 days and on day 16 CP was inoculated. Results indicated that the rats on AHE ingestion prior to CP inoculation were more resistant to CP side effects, and this might be due to endogenous antioxidant improvement by AHE polyphenols; which counteract the CP side effects. This gives an idea or approach of ingesting herbal formulations prior to chemotherapeutic agents for better protective effects.

It has been well identified that CP usage outcomes in various clinical indications, predominantly in the differentiating cells like hair follicle, bone marrow, gastrointestinal tract, testis, and ovary [46]. Treatment-associated clinical symptoms comprising; depression, fur changes, physical weakness and loss of body weight were observed in the CP group. However, these alterations were not noticed in the control and AHE-treated groups. These results suggest that AHE effectively protects against CP-induced adverse effects.

Significant alterations in the plasma lipoproteins levels were recorded in CP treated group. The high and low levels of plasma lipoproteins have a significant relation with proper liver functioning. Low density lipoproteins (LDL) and high density lipoproteins (HDL) are fundamental in lipoprotein passage. CP induced hepatic fibrosis with high cholesterol, triglycerides, LDL and decreased the level of HDL. These alterations in lipid profile indicated an association with liver and heart ailments in people with CP therapy [47]. Results showed significant amelioration of altered serum total cholesterol, triglycerides, LDL and HDL levels in AHE treatment groups, and pretreatment group showed more significant reversal compared to post treatment group. These findings exhibit the hepatoprotective potential of plant fraction. Analogous results were observed by Nora et al. while experimenting on male rats to inspect the protective effect of guabiju and red guava against CP-induced hypercholesterolemia [48]. Iliskovic and Singal pointed out that probucol achieved its preventive effect against adriamycin induced cardiomyopathy due to its lipid lowering and antioxidant properties, indicating that lipid lowing property is an important factor in achieving cardio-protective influence [47]. Presence of flavonoids in AHE might be responsible for the observed effect. The study of Pilehvar and colleagues showed that flavonoid rich grape seed oil decreased the triglycerides and cholesterol while increased the HDL content in male Wister rats [49].

CP administration significantly elevated serum concentration of ALT, AST, ALP, TSB and DB. The elevated levels of these serum biomarkers depict hepatic dysfunction, which could be an ancillary event following drug-induced liver damage with consequent enzyme leakage from the hepatocytes. These finding are in line with previous studies signifying the effect of antioxidants against CP damages [50, 51]. Kim et al. also indicated that a single dose of CP in mice can cause liver function impairments; characterized by elevation of AST and ALT activities through the underlying mechanism of CP-induced inflammation [52]. Moreover, Liao et al. demonstrated that CP altered liver function of male albino mice accompanied by significantly increased ALT activity in mice via a mechanism of oxidative stress caused by augmented MDA content and reduced GSH levels [53]. AHE protect cellular damage by decreasing the elevated levels of serum biomarkers as compared to CP alone treated group. The antioxidant and anti-inflammatory potential of AHE might be the underlying mechanism [19, 20]. Polyphenols in AHE i.e., Gallic acid and catechin might be responsible for observed effects. Our results correspond to the previous investigation revealing the reversal of increased serum enzymes in paracetamol-induced liver damage by Gallic acid [54]. This might be due to the prevention of the leakage of intracellular enzymes by its membrane-stabilizing and antioxidant activity. Biochemical observations were supported by the limited extent of histopathological lesions in AHE treatment groups.

Diminished antioxidant defense system including both enzymatic and non-enzymatic antioxidant molecules, and oxidative stress induced generation of ROS are major alterations in CP induced toxicity [55]. Amongst the cellular antioxidants SOD, POD, CAT, QR, GST, GSH, γ-GT, GPx and GR are extensively explored because of their noteworthy role in defense system. Superoxide dismutase is an exceptionally effective antioxidant enzyme that catalyzes the dismutation reaction of superoxide to H_2_O_2_ and O_2_. Whereas, catalase is a ubiquitous enzyme but mainly rich in liver further engages in breakdown of hydrogen peroxide (H_2_O_2_) to water. In the GSH (glutathione) reaction system, GSH is oxidized to GSSG by the help of GPx which consecutively converted back to GSH by the reducing power of GR. GSH also work as a cofactor for GST that is present equally in the cytosol and endoplasmic reticulum, basically involve in catalyzing the production of GSH electrophile conjugate therefore detoxify xenobiotics to produce stable compounds [56]. It is observed that lipids peroxidation can cause genetic up regulation of fibrogenic cytokines by initiating the production of collagen and stimulating hepatic stellate cells [57]. Lipid peroxidation is one of the principal causes of liver damage and its end product MDA is a major reactive aldehyde used as an oxidative stress marker [46]. In our study CP inoculation results in significant increase in lipid peroxidation; reflected by enhanced liver tissue MDA content, NO and H_2_O_2_ levels, and decrease tissue antioxidant enzymes validating oxidative stress. These findings are concurrent with previous finding regarding CP side effects; displaying marked increase in MDA levels, a lipid peroxidation biomarker, and a marked reduction in glutathione (GSH) levels in liver tissues after CP treatment indicating oxidative stress-induced hepatotoxicity [55, 58]. In the current investigation, the tissue protein content and the level of oxidative stress markers retained towards normal (control) with AHE treatment. This restoration was accompanied with improvement of antioxidant enzymes. AHE pretreatment more significantly provide protective impact as compared to AHE post administration, illustrating the preventive effect of AHE against CP induce intoxications. Analogous findings were also presented in the study of Wahhab et al. [59] while investigating the protective influence of ginseng extract against CP persuaded hepatotoxicity. AHE had a protective effect against hepatotoxicity induced by cisplatin administration to rats, and the hepatoprotective effects of AHE may be due to inhibiting oxidative stress and increasing hepatic antioxidant status. Our results are in accordance with the study of Kannan and colleagues demonstrating the preventive potential of *A. nilotica* (250 mg/kg·bw) via attenuation of alterations in serum biomarkers of liver function, elevation of GSH level and inhibition of lipid peroxidation [60].

The histological architecture is direct method for evaluating the efficacy of test samples at organ level, moreover its offers correlation amongst the activities of serum biomarkers, tissue enzyme assays and morphological alterations. Remarkable difference in liver function tests (LFTs) strongly represents the histological verification of fibrosis in liver. Fibrosis not only disturbs the normal structural pattern but also interrupts the flow of blood to prevent the delivery of nutrients to liver tissues [61]. Liver histology of CP administered group showed marked histopathological alterations in lymphocyte and kupffer cells, dissolution of hepatic cords, which give the impression of empty vacuoles aligned by strands of necrotic hepatocytes, nuclear disintegration, vacuolar degeneration, apoptotic cell death, fibrosis and collagen deposition in some parts. The liver tissue samples of plant treated groups exhibited diminished necrosis, slight inflammatory cells without damage to cell membrane, decrease fatty degenerations, cytoplasmic vacuolization, and diminished lobular necrosis around the central vein exhibit degree of assurance provided by AHE treatment. AHE provide significant protection when administered before CP injection, although AHE treatment after CP injection was also capable of prevents the CP-induced intoxication. Importantly, AHE pre-treatment was comparable to silymarin treatment groups in respective experiments, revealing its potential equivalence to silymarin for providing protection against CP induced hepatic damages respectively. The mechanism behind the hepatoprotective activity of silymarin can be explained based on its antioxidant properties due to the phenolic compounds. It also executes stimulation of liver cell regeneration and cell membrane stabilization to preclude hepatotoxic agents from pervading hepatocytes. Furthermore, it escort liver cells through wide-ranging action, comprising suppression of toxin penetration in to hepatic cells via binding to cell, improving tissue glutathione levels, impeding lipid peroxidation and augmenting hepatocyte protein synthesis [62]. Similar to silymarin, the mechanism of hepato-protection by AHE might be owing to the antioxidant and free radical quenching potential of *A. hydaspica* [55]. Catechin is the major compounds of AHE, and previous studies affirm the hepatoprotective effect of catechin against chemically induced hepatotoxicity. The mechanism of protective effect by catechin was inhibition of oxidative stress, increase antioxidant status and inhibition of apoptosis in hepatocytes [63]. Hence the presence of polyphenols in AHE might be responsible for the hepatoprotective effect by reducing oxidative stress.

## Conclusion

Our results suggest that AHE may be a useful protective agent against cisplatin-induced side effects including hepatic injury caused by oxidative stress. Amelioration of lipid profile, liver function markers in serum, attenuation of oxidative stress markers and increased activities of various antioxidant enzymes indicates that AHE is able to protect various pathological conditions including; oxidative stress and dyslipidemia. The observed protective potential might be due to the occurrence of antioxidant compounds viz. flavonoids, phenolics and tannins in AHE. The antioxidant constituents present in the AHE such as +catechin, gallic acid, methyl gallate 7-*O*-galloyl catechin might work in synergism to protect CP induced oxidative stress. However further investigations are required to unveil the precise mechanism by which *A. hydaspica* mediates its therapeutic action.
